# Bovine Lactoferricin Induces Intestinal Epithelial Cell Activation through Phosphorylation of FAK and Paxillin and Prevents Rotavirus Infection

**DOI:** 10.4014/jmb.2106.06044

**Published:** 2021-06-30

**Authors:** Ye Young Jeong, Ga Young Lee, Yung Choon Yoo

**Affiliations:** Department of Microbiology, College of Medicine, Konyang University, Daejeon 35365, Republic of Korea

**Keywords:** Lactoferricin, epithelial cells, cell adhesion, FAK, rotavirus infection, anti-viral activity

## Abstract

We investigated the effect of bovine lactoferricin (Lfcin-B), a peptide derived from bovine lactoferrin, on activation of intestinal epithelial cells in IEC-6 intestinal cell, and protection against in vivo rotavirus (RV) infection. Treatment with Lfcin-B significantly enhanced the growth of IEC-6 cells and increased their capacity for attachment and spreading in culture plates. Also, Lfcin-B synergistically augmented the binding of IEC-6 cells to laminin, a component of the extracellular matrix (ECM). In the analysis of the intracellular mechanism related to Lfcin-B-induced activation of IEC-6 cells, this peptide upregulated tyrosine-dependent phosphorylation of focal adhesion kinase (FAK) and paxillin, which are intracellular proteins associated with cell adhesion, spreading, and signal transduction during cell activation. An experiment using synthetic peptides with various sequences of amino acids revealed that a sequence of 9 amino acids (FKCRRWQWR) corresponding to 17-25 of the N-terminus of Lfcin-B is responsible for the epithelial cell activation. In an in vivo experiment, treatment with Lfcin-B one day before RV infection effectively prevented RV-induced diarrhea and significantly reduced RV titers in the bowels of infected mice. These results suggest that Lfcin-B plays meaningful roles in the maintenance and repair of intestinal mucosal tissues, as well as in protecting against intestinal infection by RV. Collectively, Lfcin-B is a promising candidate with potential applications in drugs or functional foods beneficial for intestinal health and mucosal immunity.

## Introduction

The gastrointestines can be described as epithelial-lined mucosal tissues that play an important role in nutrient absorption and host defense systems [1]. Interactions between enterocytes and the basal membrane are essential for cell adhesion, migration, differentiation, and maintenance of mucosal tissue integrity [2, 3]. Cell adhesion triggers migration, proliferation, and differentiation [4, 5], while the binding of cell surface molecules like integrin to the extracellular matrix (ECM) increases tyrosine phosphorylation of several focal adhesion proteins such as FAK (125 kDa) and paxillin (68 kDa) to bring about the assembly of actin filaments as well as cell proliferation [6, 7].

Rotavirus (RV) is a major agent of viral gastroenteritis in various animal species. It is regarded as the leading cause of acute diarrhea in human infants and young children under the age of five and annually causes 215,000 deaths worldwide [8, 9]. Thus, the mortality and morbidity of RV-associated diarrhea makes this virus an important pathogenic agent to be controlled. Current live vaccines can induce anti-diarrheal effect, but they are neither distributed around the world nor sufficiently effective in some developing countries [10]. Therefore, the development of alternative approaches to prevent RV-induced diarrhea is urgently needed. Since intestinal epithelial cells (IECs) are the primary responder to RV in the initial period of infection, the dysfunction of these cells has been considered as a major cause of diarrhea [11].

Breast milk has a lot of components that take charge of diverse physiological properties, such as nutritional source and host defense against pathogens, especially on mucosal surfaces of the gastrointestinal tract [12]. The iron-binding protein lactoferrin (LF), mainly found in body fluids like breast milk, is well known to have a diversity of biological functions, *i.e.*, antimicrobial activity, regulation of immune responses [13, 14] and transcriptional activation of cells [15]. Upon ingestion of LF, the protein is degraded by gastrointestinal enzymes in the stomach, like pepsin, and may create bioactive peptides like lactoferricin (Lfcin-B) [16, 17]. Lfcin-B has been shown to be a key peptide responsible for the antibacterial activity of bovine LF [18, 19]. Taken together, it is possible that Lfcin-B generated from pepsin lysate of LF arrives at the intestines, where it interacts with IECs to modulate cell functions such as anti-viral activity against RV. However, as of yet, there has been no report on the biological actions of Lfcin-B on IECs and RV infection.

In the present study, we suggest that Lfcin-B induces the activation of IEC-6 intestinal epithelial cells via the phosphorylation of FAK and paxillin, and effectively prevents RV infection in in vivo experiments.

## Materials and Methods

### Cell Culture and Virus

Rat small intestinal crypt cell line (IEC-6) was cultivated in Dulbeccós Modified Eaglés Medium (DMEM) supplemented with 5 % (v/v) heat-inactivated fetal bovine serum, 4 mM L-glutamine, 110 mg/l sodium pyruvate, 20 mM sodium bicarbonate, 10 mg/ml gentamicin, 0.1 IU/ml insulin at 37°C and 5% CO_2_. MA-104 cells were maintained in Eagle’s minimum essential medium (EMEM) supplemented with 5 mM glutamine, 0.1% sodium bicarbonate, 50 µg/ml gentamicin, 3 µg/ml amphotericin B, and 10% fetal calf serum. RV (simian rotavirus SA11; RV-SA11) was kindly provided by Dr. J. Arikawa (Hokkaido University, Japan). The titer of virus stocks used in these experiments was 2.5 × 10^8^ plaque-forming units (PFU)/ml [20].

### Animals

Specific pathogen-free pregnant Balb/c mice were obtained from Raon Bio Ltd. (Korea). The mice had pelleted diets and water ad libitum. Animal experiments were performed according to the Laboratory Animal Control Guidelines of IACUC of Konyang University (Approval No. P-16-01-A-01).

### Antibodies and Reagents

Mouse anti-rat actin antibody and anti-phosphotyrosine monoclonal antibody conjugated to horseradish peroxidase (HRP) were purchased from Thermo Fisher Scientific (USA). Antibodies specific to FAK (clone 77) and paxillin (clone 349) were purchased from Transduction Laboratories (USA). The secondary antibody used for immunoblotting, anti-mouse IgG(H+L) F(ab')_2_ conjugated to HRP, was purchased from Southern Biotech (USA). Protein G-Sepharose beads used for immunoprecipitation were obtained from Pharmacia Biotech (Japan). Purified Lfcin-B (amino acid sequence; FKCRRWQ WRMKKLGAPSIT CVRRAF), a cationic peptide corresponding to residues 17-41 near the N-terminus of bovine LF [21], was kindly donated by the Food Research and Development Laboratory (Morinaga Milk Industry Co., Ltd., Japan). Peptides with partial sequences of Lfcin-B were supplied by Cosmo Genetech Co. (Korea). The other reagents except those otherwise indicated were purchased from Sigma Chemical Co. (USA).

### Cell Proliferation Assay

The proliferation assay was carried out as described previously [22] with some modifications. IEC-6 cells (1×10^4^ cells/ml) were treated with the indicated doses of Lfcin-B and various types of peptides or bovine serum albumin (BSA) in 96-well microculture plates for 48 h. The cells were pulsed with tritiated thymidine ([^3^H]-TdR, 0.5 mCi/well, Amersham International, UK) for 8 h. Then, the cells were harvested using a Filtermate 196 (Thermo Fisher Scientific) and the radioactivity was measured in a Matrix 96 direct beta counter (Thermo Fisher Scientific). The radioactivity was expressed as counts per minute (CPM) of mean ± SD. The number of IEC-6 cells incubated for the indicated times was calculated by the method of trypan blue dye exclusion.

### Cell Attachment and Spreading Assay

Cell attachment and spreading were assayed according to the method described previously [23] with some modifications. In the cell attachment assay, IEC-6 cells (1 × 10^5^/well) were cultured with the indicated doses of Lfcin-B in the presence or absence of laminin (LN, 1 µg/ml) in 24-well culture plates for 30 min. Bovine serum albumin (BSA) was used as a negative control protein. After removing non-adherent cells by washing with warm (37°C) PBS, adherent cells were stained with crystal violet solution (0.5% crystal violet in 20% methanol) at room temperature for 30 min. Thereafter, the cells were lysed by 100 µl/well of 30% acetic acid, and optical density (OD) was measured at 595 nm. For the cell spreading assay, IEC-6 cells were incubated with 50 µg/ml of Lfcin-B or BSA for various times (30, 60, and 90 min) in 6-well culture plates, and the number of spreading cells in a distinct microscopic area was counted according to the criteria described previously [24]. The percentage of spreading cells in total was calculated from three individuals counting two hundred cells in each well under an inverted microscope (CKX41, Olympus, Japan).

### Immunoprecipitation and Immunoblotting

IEC-6 cells (1 × 10^6^/well) were treated with or without Lfcin-B (50 µg/ml) in 6-well plate for the indicated times. Cells were washed with ice-cold PBS and scraped in ice-cold lysis buffer (1 % NP-40, 0.5 % DOC, 0.1 % SDS, 1 mM EDTA, 20 mM NaF, 1 mM sodium vanadate, 10 mg/ml aprotinin, 0.5 mM benzamidine, 5 mg/ml leupeptin, 1 mM phenyl-methanesulphonyl fluoride). The proteins (30 µg) of cell lysates were reacted with anti-paxillin or anti-FAK antibody at 37°C for 2 h, followed by immunoprecipitating with 20 µl of 50% Protein G-Sepharose beads in the same conditions on a rotating shaker. The immunocomplexes were washed 5 times with 1 ml of cold PBS, re-suspended in 20 µl of electrophoresis sample buffer, and boiled for 10 min. Proteins (5 µg/lane) were developed on 8% SDS-PAGE gels, transferred to PVDF membranes (Micron Separations., USA), and probed with the appropriate antibody. PVDF membranes were blocked with 1% BSA in Tris-buffered saline/Tween-20 (TBS-T, 10 mM Tris base, pH 7.4, 100 mM NaCl, 0.1% Tween-20), then reacted with anti-FAK or anti-paxillin antibody (1:1000) dissolved in TBS-T for 1 h, followed by incubation with the HRP-conjugated anti-mouse IgG antibody (1:3500) for 1 h. For detection of phosphorylated FAK and paxillin, PVDF membranes containing the immunocomplexes were incubated with a strip buffer (100 mM 2-mercaptoethanol, 2% SDS, 62.5 mM Tris/HCl, pH 6.7) at 60°C for 1 h. After washing 5 times with PBS, the blots were blocked with 1% BSA in TBS-T, incubated with anti-phosphotyrosine antibody (1:5000) for 1 h, and reacted with HRP-conjugated secondary antibody. Immunoreactive bands were visualized using a Bioanalytical Imaging System c300 (Azure Biosystems, USA).

### RV Infection Experiment

RV (1.5 × 10^6^ PFU/50 µl/mouse) was inoculated *per os* (p.o.) into the groups of seven 10-day-old Balb/c newborn mice. Mice were fasted for 4 h before RV infection [25]. Lfcin-B was administered p.o. 1 day before virus infection. Ginsenoside Rb2 was used as a positive control for protection against RV [20]. A clinical score of diarrhea induced by RV infection was determined by individual severity of diarrhea every 24 h after virus infection: point 2; serious, point 1; moderate, point 0; cured. The diarrhea score for each group was calculated as follows: (the number of mice under serious diarrhea) × 2 + (the number of mice under moderate diarrhea) × 1/total number of mice [25]. The total diarrhea score was estimated as cumulative diarrhea scores obtained during the entire observation time.

### RV Isolation from the Bowels

Virus titers in the bowels of RV-infected newborn mice were determined by plaque formation on MA-104 cells as described previously [20]. Briefly, the bowels removed from the mice were homogenized in 1 ml of EMEM. After centrifugation, the supernatants were massed up to 5 ml with EMEM, and stored at -80°C until use. Each homogenate (0.5 ml/well) in 1,000-fold dilution was added onto the monolayer of MA-104 cells in 6-well culture plates and incubated at 37°C for 1 h. After washing twice with EMEM, the cells were overlaid with 2.5 ml of EMEM containing 0.7% purified agar (Agarose; SeaKem ME, FMC BioProducts, USA) and 0.0001% trypsin, and incubated at 37°C for 5 days. The cells were subsequently overlaid with EMEM (2 ml/well) containing 0.7%purified agar and 0.005% neutral red for 2 days. Thereafter, the plaques formed in RV-infected MA-104 cells were counted.

### Statistical Analysis

The statistical significance was determined by Student's two-tailed *t*-test.

## Results and Discussion

### Effect of Lfcin-B on Cell Proliferation and Growth

**Fig. 1:**
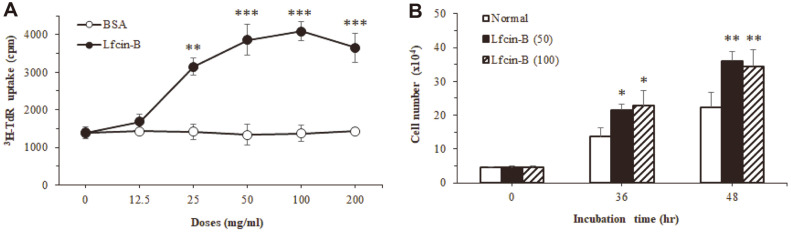
Effect of Lfcin-B on the proliferation of IEC-6 epithelial cells. IEC-6 cells were incubated with various doses of Lfcin-B or BSA for 48 h at 37°C. The proliferation of IEC-6 cells was measured by DNA synthesis using [^3^H]-TdR uptake assay. Cell number was calculated by trypan blue dye exclusion. These results are representative of three independent experiments. *p < 0.05, **p < 0.01, ***p < 0.001, compared with control (BSA-treated) group (by Student’s two-tailed *t*-test).

IEC-6 cell, a non-transformed rat jejunum crypt cell line [26], has been widely employed in studies on the effect of various growth factors and cytokines on intestinal crypt cell growth and maturation [27, 28]. To address the influence of Lfcin-B on the proliferation of intestinal cells, we examined its activity to enhance DNA synthesis of IEC-6 cells by [^3^H]-TdR uptake assay. Treatment with Lfcin-B significantly increased DNA synthesis of IEC-6 cells in a dose-dependent manner, showing maximal activity from the dose of 50 µg/ml (Fig. 1A). However, BSA, a control protein, had no effect. When we examined the number of IEC-6 cells at incubation times of 36 and 48 h, the cells treated with Lfcin-B (50 or 100 µg/ml) showed a significant increase in cell number (Fig. 1B) from the incubation of 36 h. The results indicated that Lfcin-B actively augments the growth of IECs through acceleration of DNA synthesis.

### Effect of Lfcin-B on Attachment and Spreading of IEC-6 Cells

**Fig. 2:**
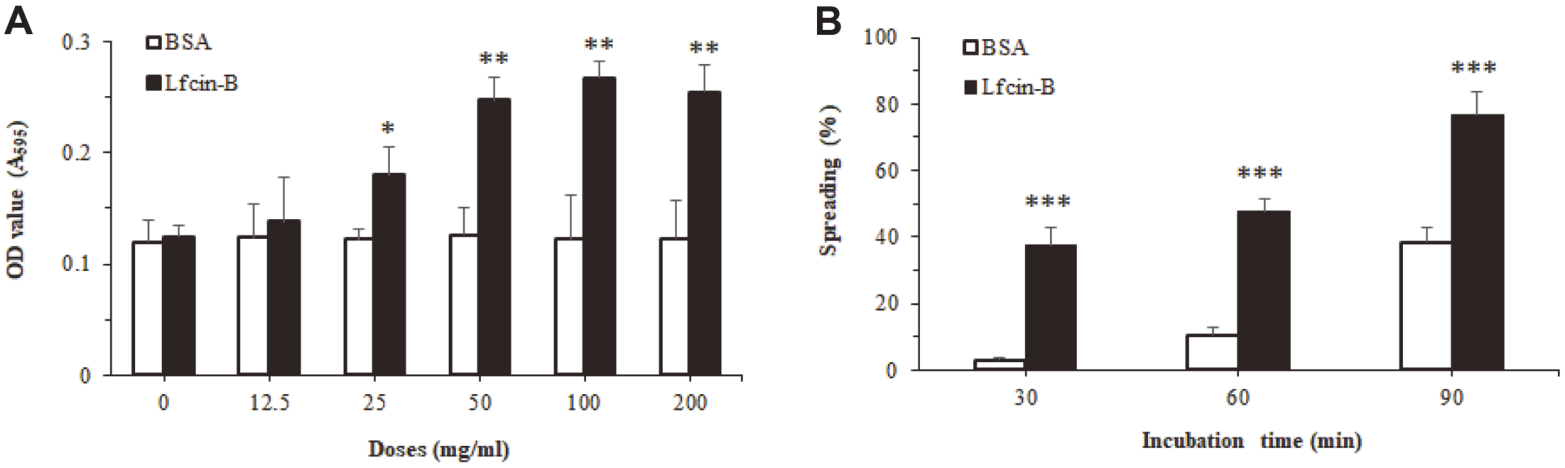
Effect of Lfcin-B on cell adhesion and spreading of IEC-6 epithelial cells. Cell adhesion (**A**) was measured using IEC-6 cells treated with the indicated doses of Lfcin-B or BSA at the incubation time of 30 min, and cell spreading (**B**) was assayed 30, 60 and 90 min after Lfcin-B (50 μg/ml) treatment. These results are representative of three (**A**), or two (**B**) different experiments, respectively. *p < 0.05, **p < 0.01, ***p < 0.001, compared with control (BSA-treated) group (by Student’s twotailed *t*-test).

**Fig. 3:**
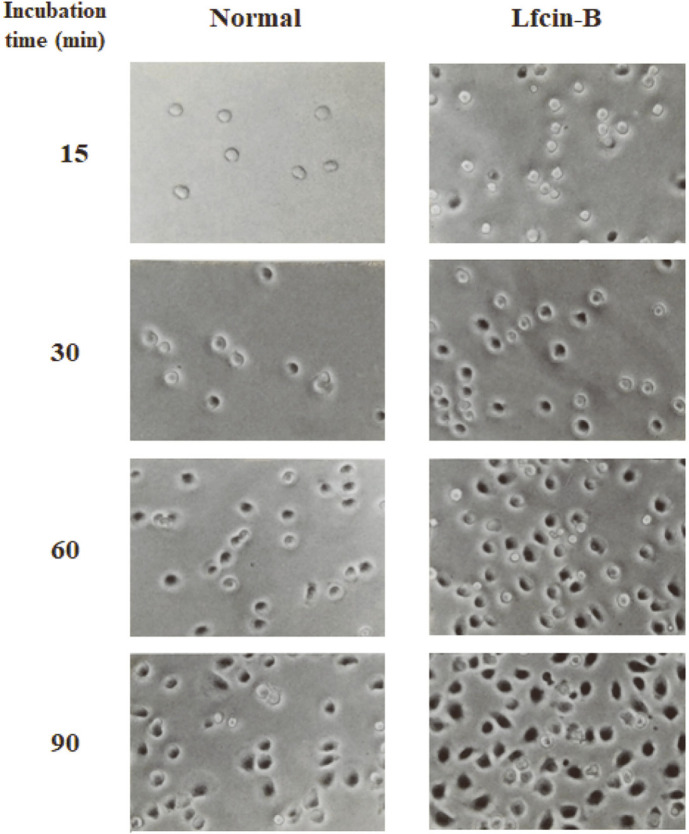
Inverted microphotograph of IEC-6 cells treated with Lfcin-B. The cells were incubated in the presence of Lfcin-B (50 μg/ml), and inverted microphotographs of adherent cells were visualized using an inverted microscope at the incubation times of 15, 30, 60, and 90 min. The photos were taken at 400× magnification.

Cell adhesion is a multistep process involving primary receptor-ligand interactions followed by secondary events that may lead to the formation of focal contacts and the expression of cell functions [29]. Generally, focal adhesion (attachment) is an important event during structural links between the cytoskeleton and extracellular matrix (ECM), and induction of signal transduction in adherent cells like IECs. To examine the influence of Lfcin-B on attachment of IECs, IEC-6 cells were treated with various doses of Lfcin-B for 30 min, and attached cells were estimated using cell staining with crystal violet. IEC-6 cells treated with Lfcin-B showed significantly higher attachment than that of negative control (BSA-treated) cells, and its enhancing effect was dose-dependent (Fig. 2A). Maximal activity of Lfcin-B to enhance cell attachment was also observed from the dose of 50 µg/ml. Next, to elicit the effect of Lfcin-B on spreading of IECs, IEC-6 cells were incubated in the presence of Lfcin-B (50 µg/ml) or BSA as a negative control, and the spreading cells were examined at the incubation times of 30, 60 and 90 min. As shown in Fig. 2B, spontaneous spreading of IEC-6 cells increased with the lapse of incubation time. Meanwhile, the cells treated with Lfcin-B showed a significant increase of cell spreading at all incubation times. In addition, an inverted photomicrograph of cultured cells clearly showed that Lfcin-B promoted cell adhesion and spreading even at the early period (15 min) of incubation time (Fig. 3).

**Fig. 4:**
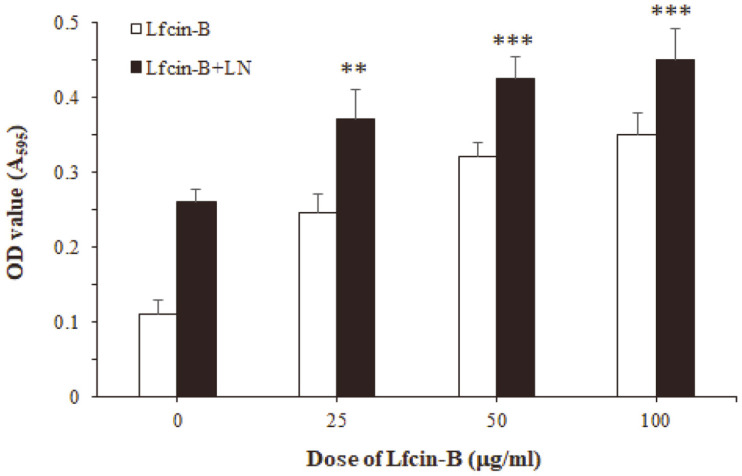
Effect of Lfcin-B on cell adhesion of IEC-6 cells in combination with LN. The cells were incubated with the indicated doses of Lfcin-B in culture plate coated with LN (1 μg/ml) for 30 nin. **p < 0.01, ***p < 0.001, compared with Lfcin-Btreated group (by Student’s two-tailed *t*-test).

The epithelial basement membranes (BM) are ECM structures consisting of well-organized glycoproteins, particularly laminin (LN), fibronectin, and type IV collagen. These proteins regulate biological processes such as migration, proliferation, and differentiation of IECs [30]. Among ECM molecules, LN, one of the most abundant proteins present in the BM, can bind to IECs and modulate several biological functions of the cells, including cell growth, cell adhesion and migration, and gene expression [31]. When IEC-6 cells were incubated with Lfcin-B in LN-coated culture plates, treatment with Lfcin-B synergistically enhanced cell adhesion of the cells in a dose-dependent manner (Fig. 4). This finding indicated that Lfcin-B can upregulate the biological and physiological functions of epithelial cells by itself as well as in combination with ECM molecules like LN.

The intestinal epithelium is sometimes damaged by toxins or pathogenic microorganisms, and, in this case, mucosal restitution rapidly works to repair the damaged epithelium. This restoration work includes the peeling of damaged epithelial cells and the spreading and migration of viable cells to reconstruct epithelial continuity [32]. The intestinal mucosa is established by epithelial cells that become differentiated after migrating from the crypts to the villus. This means that activation of intestinal crypt cells may be directly connected to upregulation of maintenance and repair of intestinal mucosa [26]. The data from Fig. 1 to Fig. 4 consistently proved that Lfcin-B enhanced not only cell proliferation but also attachment and spreading of the intestinal crypt cell line, IEC-6. These results suggest that Lfcin-B can play roles in maintenance of the mucosal lining and repair of the damaged epithelium in the intestines.

### Lfcin-B Induces Phosphorylation of FAK and Paxillin

**Fig. 5:**
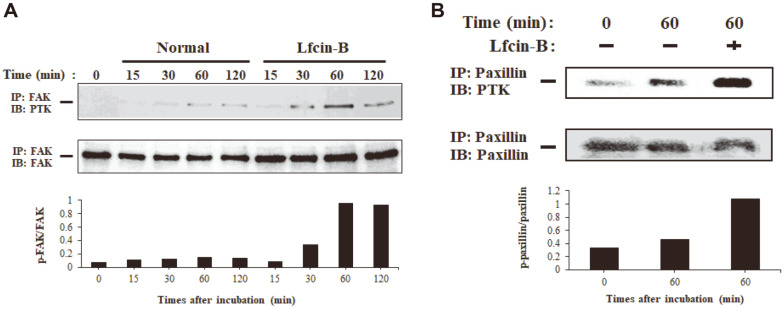
Effect of Lfcin-B on phosphorylation of FAK in IEC-6 cells. Phosphorylation of FAK in IEC-6 cells treated with Lfcin-B (50 μg/ml) was measured by immunoprecipitation and western blot assays by the method described in Materials and Methods. PTK is an abbreviation for protein tyrosine kinase. Bar graphs represent the results of densitometry analysis.

Focal adhesion is a highly dynamic membrane-associated multi-protein structure that binds cells to the ECM and regulates various cellular and biological actions. FAK and paxillin are potential regulatory molecules involved in interactions between cellular proteins and extracellular molecules during focal adhesion and have been recognized as a critical link in the signal transduction pathway in adherent cells [6, 33]. To examine whether the ability of Lfcin-B to activate IEC-6 cells is related to induction of focal adhesion in the cells, we examined phosphorylation of FAK and paxillin by Lfcin-B using an immunoprecipitation assay, in which cellular proteins were precipitated with anti-paxillin or anti-FAK antibody followed by immunoblotting with anti-phosphtyrosine antibody. Lfcin-B treatment prominently augmented tyrosine phosphorylation of FAK in IEC-6 cells, showing apparent enhancement from the early incubation period of 15 min and maximal effect at 60 min (Fig. 5A). Furthermore, IEC-6 cells treated with Lfcin-B indicated higher level of phosphorylated paxillin than that of untreated cells at 60 min (Fig. 5B). These results suggest that the activity of Lfcin-B to enhance attachment and spreading of intestinal IEC-6 epithelial cells is associated with phosphorylation of FAK and paxillin. Considering that, in general, the event of phosphorylation of intracellular proteins such as FAK and paxillin in adherent cells occurs during interactions with extracellular proteins like those of the ECM (Kolachala *et al*., 2007), it is significant that Lfcin-B acts as an inducer of cellular signals to result in cell proliferation and adhesion.

### Minimal Sequence of Lfcin-B Responsible for Activation of IEC-6 Cells

**Fig. 6:**
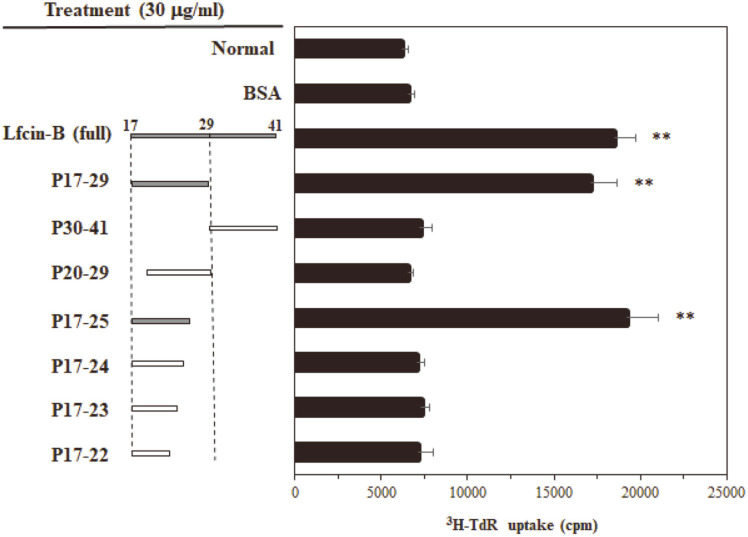
Determination of minimal sequence of Lfcin-B responsible for IEC-6 cell activation. IEC-6 cells were incubated with Lfcin-B or various sequences of this peptide (30 μg/ml) for 48 h. Cell proliferation was determined by [^3^H]-TdR uptake assay. **p < 0.001, compared with control (BSA-treated) group (by Student’s two-tailed *t*-test).

Next, to determine the minimal structure of Lfcin-B responsible for its ability to induce IEC-6 cell activation, we synthesized many types of peptides containing the partial sequences of Lfcin-B, and investigated their ability to enhance cell proliferation. As seen in Fig. 6, the minimal sequence showing identical activity of the full length of Lfcin-B was 9 amino acids (FKCRRWQWR; P17-25). This result means that the nine amino-acid peptide from the N-terminus of Lfcin-B is a minimal peptide of Lfcin-B responsible for the activation of IEC-6 epithelial cells.

The present study demonstrated for the first time that Lfcin B, a peptide derived from the milk protein lactoferrin, is a novel peptide that induces activation of IEC-6 rat intestinal epithelial cells through phosphorylation of FAK and paxillin, and the minimal sequence responsible for its activity is FKCRRWQWR, which corresponds to nine amino acids from the N-terminus of Lfcin-B. These findings suggest that Lfcin-B is a biologically active milk protein-derived peptide that contributes to recovery of damaged intestinal epithelium and enhancement of host defense system in the gut.

### Effect of Lfcin-B on RV Infection

**Fig. 7:**
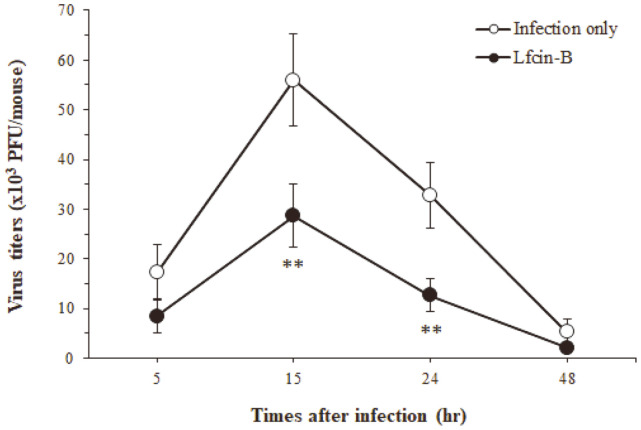
Inhibitory effect of Lfcin-B on virus growth in the bowels of RV-infected newborn mice. Balb/c newborn mice were administered orally with Lfcin-B (50 μg/mouse) 1 day before RV infection. Virus titers in the bowels were measured by counting the number of PFU on MA-104 cells as described in Materials and Methods. **p < 0.01, compared with non-treated (infection only) group (by Student's two-tailed *t*-test).

Considering that IECs react first to RV at the onset of infection [11], the condition of the cells may be a very important factor influencing the occurrence of RV-induced acute diarrhea. Based on the results showing that Lfcin-B can activate IECs, we examined whether this peptide prevents the intestinal infection caused by RV. Oral administration of Lfcin-B 1 day prior to infection resulted in a highly protective effect against RV-induced acute diarrhea (Table 1). The reason was unclear, but a lower dosage of Lfcin-B (50 µg) was more effective than a higher dosage (100 µg). RV is a virus that infects IECs at the initial stage of infection, proliferates, and then causes acute diarrhea [34]. Since it is important to prevent the growth of the virus in the early period of infection, we next investigated whether oral administration of Lfcin-B (50 µg) inhibits the intestinal proliferation of RV. In the newborn mouse infection model, RV showed the highest virus titer 15 h after infection, and then gradually decreased (Fig. 7). Oral administration of Lfcin-B (50 µg) significantly reduced RV titer in the intestines at least 15 h after infection. These results suggest that Lfcin-B has an inhibitory effect on RV infection and the antiviral effect of this peptide is due to suppression of the infection in epithelial cells at the initial stage of infection.

In this study, we demonstrated that Lfcin-B, a peptide derived from bovine lactoferrin, can activate IECs via upregulation of phosphorylation of FAK and paxillin, in in vitro experiments, and oral administration of this peptide also effectively prevented severe infection by RV in in vivo models. Taken together, it is strongly suggested that Lfcin-B is a potent stimulant to potentiate biological functions of the intestinal epithelial cells and non-specific resistance against RV infection. RV infection is inhibited by immunological factors such as interferons produced by immune cells in the intestinal epithelial tissue; however, it remains unclear whether Lfcin-B-induced epithelial cell activation elicits the immunological response necessary to suppress RV virus infection. Currently, research is underway to elucidate the correlation between Lfcin-B-induced epithelial cell activation and its protective activity against RV infection.

## Figures and Tables

**Table 1:** Protective effect of Lfcin-B on RV infection in newborn mice.

Treatment	Duration of diarrhea (days)		Total diarrhea score (Inhibition %)

	Total	Severit	
Infection only	2-5	2-4	5.25
Lfcin-B (50 µg)	3-4	-	1.95 (62.9)
Lfcin-B (100 µg)	2-4	2-3	2.95 (43.8)
Ginsenoside Rb2	2-4	2-3	2.25 (57.1)

Balb/c newborn mice were inoculated with RV-SA11 and administered p.o. with 50 or 100 μg of Lfcin-B one day before virus infection. Ginsenoside Rb2 (100 μg), a positive control, was administered 1, 2 and 3 days before virus infection.

## References

[1] Thompson CA, DeLaForest A, Battle MA (2018). Patterning the gastrointestinal epithelium to confer regional-specific functions. Dev. Biol..

[2] Sumagin R, Parkos CA (2015). Epithelial adhesion molecules and the regulation of intestinal homeostasis during neutrophil transepithelial migration. Tissue Barriers.

[3] Canonici A, Siret C, Pellegrino E, Pontier-Bres R, Pouyet L, Montero MP (2011). *Saccharomyces boulardii* improves intestinal cell restitution through activation of the α2β1 integrin collagen receptor. PLoS One.

[4] Kolachala VL, Bajaj R, Wang L, Yan Y, Ritzenthaler JD, Gewirtz AT (2007). Epithelial-derived fibronectin expression, signaling, and function in intestinal inflammation. J. Biol. Chem..

[5] Kopf A, Sixt M (2019). Gut homeostasis: active migration of intestinal epithelial cells in tissue renewal. Curr. Biol..

[6] Walsh MF, Ampasala DR, Rishi AK, Basson MD (2009). TGF-β1 modulates focal adhesion kinase expression in rat intestinal IEC-6 epithelial cells via stimulatory and inhibitory Smad binding elements. Biochim. Biophys. Acta..

[7] Efstathiou JA, Pignatelli M (1998). Modulation of epithelial cell adhesion in gastrointestinal homeostasis. Am. J. Pathol..

[8] Desselberger U (Rotaviruses). 2014. Virus Res..

[9] Clark A, Black R, Tate J, Roose A, Kotloff K, Lam D (2017). Estimating global, regional and national rotavirus deaths in children aged < 5 years: current approaches, new analyses and proposed improvements. PLoS One.

[10] Jiang V, Jiang B, Tate J, Parashar UD, Patel MM (2010). Performance of rotavirus vaccines in developed and developing countries. Hum. Vaccin..

[11] Rollo EE, Kumar KP, Reich NC, Cohen J, Angel J, Greenberg HB (1999). The epithelial cell response to rotavirus infection. J. Immunol..

[12] Lee J, Yoo YC (2006). Determination of the minimal sequence of bovine lactoferricin responsible for apoptosis induction in THP-1 cells. Lab. Anim. Res..

[13] Kruzel ML, Zimecki M, Actor JK (2017). Lactoferrin in a context of inflammation-induced pathology. Front. Immunol..

[14] Legrand D (2016). Overview of Lactoferrin as a natural immune modulator. J. Pediat..

[15] He J, Furmanski P (1995). Sequence specificity and transcriptional activation in the binding of lactoferrin to DNA. Nature.

[16] Furlund CB, Ulleberg EK, Devold TG, Flengsrud R, Jacobsen M, Sekse C (2013). Identification of lactoferrin peptides generated by digestion with human gastrointestinal enzymes. J. Dairy Sci..

[17] Miyauchi H, Kaino A, Shinoda I, Fukuwatari Y, Hayasawa H (1997). Immunomodulatory effect of bovine lactoferrin pepsin hydrolysate on murine splenocytes and Peyer's Patch cells. J. Dairy Sci..

[18] Bellamy W, Takase M, Yamaguchi K, Wakabayashi H, Kawase K, Tomita M (1992). Identification of the bactericidal domain of lactoferrin. Biochem. Biophys. Acta.

[19] Yamauchi K, Tomita M, Giehl TJ, Ellison RT (1993). Antibacterial activity of lactoferrin and a pepsin-derived lactoferrin peptide fragment. Infect. Immun..

[20] Yang H, Oh KH, Kim HJ, Cho YH, Yoo YC (2018). Ginsenoside-Rb2 and 20(S)-Ginsenoside-Rg3 from Korean red ginseng prevent rotavirus infection in newborn mice. J. Microbiol. Biotechnol..

[21] Yoo YC, Watanabe R, Koike Y, Mitobe M, Shimazaki K, Watanabe S (1997). Apoptosis in human leukemic cells induced by lactoferricin, a bovine milk protein-derived peptide: involvement of reactive oxygen species. Biochem. Biophys. Res. Commun..

[22] Sato K, Yoo YC, Mochizuki M, Saiki I, Takahashi T, Azuma I (1995). Inhibition of tumor-induced angiogenesis by a synthetic lipid A analogue with low endotoxicity, DT-5461. JPN. J. Cancer Res..

[23] Ray RM, Viar MJ, McCormack SA, Johnson LR (2001). Focal adhesion kinase signaling is decreased in polyamine-depleted IEC-6 cells. Am. J. Physiol. Cell Physiol..

[24] Timer J, Chen YQ, Liu B, Bazaz R, Tayor JD, Honn KV (1992). The lipoxygenase metabolite 12(S)‐hete promotes α_llb_β_3_integrin‐mediated tumor‐cell spreading on fibronectin. Int. J. Cancer.

[25] Fukushima A, Yoo YC, Yoshomatsu K, Matsuzawa K, Tamura M, Tono-oka S (1996). Effect of MDP-Lys(L18) as a mucosal immunoadjuvant on protection of mucosal infections by Sendai virus and rotavirus. Vaccine.

[26] Chen J, Zhang R, Wang J, Yu P, Liu Q, Zeng D (2015). Protective effects of baicalin on LPS-induced injury in intestinal epithelial cells and intercellular tight junctions. Can. J. Physiol. Pharmacol..

[27] Ouko L, Ziegler TR, Gu LH, Eisenberg LM, Yang VW (2004). Wnt11 signaling promotes proliferation, transformation, and migration of IEC6 intestinal epithelial cells. J. Biol. Chem..

[28] Pearce SC, Coia HD, Karl JP, Pantoja-Feliciano IG, Zachos NC, Racicot K (2018). Intestinal in vitro and ex vivo models to study hostmicrobiome interactions and acute stressors. Front. Physiol..

[29] Santos MF, Viar MJ, McCormack S A, Johnson JR (1997). Polyamines are important for attachment of IEC-6 cells to extracellular matrix. Am. J. Physiol..

[30] Vllasaliu D, Falcone FH, Stolnik S, garnett M (2014). Basement membrane influences intestinal epithelialcell growth and presents a barrier to the movementof macromolecules. Exp. Cell Res..

[31] Teller IC, Beaulieu JH (2001). Interactions between laminin and epithelial cells in intestinal health and disease. Expert. Rev. Mol. Med..

[32] Chang RM, Wen LQ, Chang JX, Fu YR, Jiang ZP, Chen S (2013). Repair of damaged intestinal mucosa in a mouse model of sepsis. World J. Emerg. Med..

[33] Devedec SEL, Geverts B, Bont H, Yan K, Verbeek FJ, Houtsmuller AB (2012). The residence time of focal adhesion kinase (FAK) and paxillin at focal adhesions in renal epithelial cells is determined by adhesion size, strength and life cycle status. J. Cell Sci..

[34] Yoo YC, Lee J, Park SR, Nam KY, Cho YH, Choi JE (2013). Protective effect of ginsenoside-Rb2 from Korean red ginseng on the lethal infection haemagglutinating virus of Japan in mice. J. Ginseng Res..

